# Expression of Concern

**DOI:** 10.1111/adb.13437

**Published:** 2024-09-05

## Abstract

A. Ghaderi, H.R. Banafshe, N. Mirhosseini, M, Motmaen, F. Mehrzad, F. Bahmani, E. Aghadavod, M.A. Mansournia, R.J. Reiter, M‐A. Karimi, and Z. Asemi, “The Effects of Melatonin Supplementation on Mental Health, Metabolic and Genetic Profiles in Patients Under Methadone Maintenance Treatment,” *Addiction Biology* 24, no. 4 (2019): 754‐764, https://doi.org/10.1111/adb.12650.

This Expression of Concern is for the above article, published online on 27 June 2018 in Wiley Online Library (wileyonlinelibrary.com), and has been published by agreement between the journal Editor‐in‐Chief, Rainer Spanagel, Society for the Study of Addiction, and John Wiley & Sons Ltd. The Expression of Concern has been agreed due to concerns raised regarding the integrity of the research and discrepancies in reporting. An investigation has been conducted by the National Committee for Ethics in Biomedical Research Iran, in coordination with Kashan University of Medical Sciences (KAUMS). However, without the verification of clinical records there remain sufficient doubts about the feasibility and integrity of the research undertaken. As a result, the journal has decided to issue an Expression of Concern to alert readers.

